# De-noising with a SOCK can improve the performance of event-related ICA

**DOI:** 10.3389/fnins.2014.00285

**Published:** 2014-09-19

**Authors:** Kaushik Bhaganagarapu, Graeme D. Jackson, David F. Abbott

**Affiliations:** ^1^The Florey Institute of Neuroscience and Mental Health, Austin Hospital, The University of MelbourneMelbourne, VIC, Australia; ^2^Department of Medicine, The University of MelbourneMelbourne, VIC, Australia; ^3^Department of Radiology, The University of MelbourneMelbourne, VIC, Australia

**Keywords:** functional magnetic resonance imaging (fMRI), independent component analysis (ICA), automated classification, artifacts, denoising, filter, event related ICA, Benign epilepsy with centro-temporal spikes (BECTS)

## Abstract

Event-related ICA (eICA) is a partially data-driven analysis method for event-related fMRI that is particularly suited to analysis of simultaneous EEG-fMRI of patients with epilepsy. EEG-fMRI studies in epileptic patients are typically analyzed using the general linear model (GLM), often with assumption that the onset and offset of neuronal activity match EEG event onset and offset, the neuronal activation is sustained at a constant level throughout the epileptiform event and that associated fMRI signal changes follow the canonical HRF. The eICA method allows for less constrained analyses capable of detecting early, non-canonical responses. A key step of eICA is the initial deconvolution which can be confounded by various sources of structured noise present in the fMRI signal. To help overcome this, we have extend the eICA procedure by utilizing a fully standalone and automated fMRI de-noising procedure to process the fMRI data from an EEG-fMRI acquisition prior to running eICA. Specifically we first apply ICA to the entire fMRI time-series and use a classifier to remove noise-related components. The automated objective de-noiser, “Spatially Organized Component Klassificator” (SOCK) is used; it has previously been shown to distinguish a substantial fraction of noise from true activation, without rejecting the latter, in resting-state fMRI. A second ICA is then performed, this time on the event-related response estimates derived from the denoised data (according to the usual eICA procedure). We hypothesize that SOCK + eICA has the potential to be more sensitive than eICA alone. We test the effectiveness of SOCK by comparing activation obtained in an eICA analysis of EEG-fMRI data with and without the use of SOCK for 14 patients with rolandic epilepsy who exhibited stereotypical IEDs arising from a focus in the rolandic fissure.

## 1. Introduction

Event-related functional magnetic resonance imaging (fMRI) is an MRI technique that can be used to detect changes in the Blood Oxygen Level Dependent (BOLD) hemodynamic response to neural activity in response to certain events. The conventional method for detecting event-related responses in fMRI consists of modeling the expected fMRI response to an event by convolving a stimulus presentation time-course with an assumed canonical Haemodynamic Response Function (HRF) and using linear regression to identify voxels with a significant correlation to this expected response (Josephs et al., 1997). One typically assumes that the onset and offset of neuronal activity match stimuli onset and offset, the neuronal activation is sustained at a constant level throughout the stimulus and that evoked fMRI signal changes follow the canonical HRF.

There are instances, however, when these assumptions may not be satisfied. An example is interictal epileptiform discharges (IEDs), which are pathological patterns of activity generated by the brain of patients with epilepsy between seizures (de Curtis et al., 2012). IEDs produce marked and stereotyped trace deviations on electroencephalography (EEG) recordings and can be studied using fMRI by using a simultaneous acquisition of EEG (EEG-fMRI) in order to identify the event timings (Lemieux et al., 2001; Bnar et al., 2002). Studies have shown that the onset of the neuronal activity underlying the EEG discharge may not always coincide with the EEG onset (Bai et al., 2010; Carney et al., 2010; Masterton et al., 2010). For example, Carney et al. (2010) identified changes in BOLD signal which precede the onset of epileptiform activity. In addition, it is also reported that the use of the same HRF in all patients may not be appropriate and that individual-based HRF models provide increases in extent and degree of activation (Masterton et al., 2010; Storti et al., 2013).

To address the above issues, we developed an algorithm, dubbed event-related independent components analysis (eICA), which allowed for less constrained analyses capable of detecting early, non-canonical responses (Masterton et al., 2013a,b). Event-related ICA is a technique that provides an estimate of the underlying components that give rise to the observed event-related fMRI signal changes throughout the brain, and importantly, does not rely upon the specification of an HRF model or predefined Regions of Interest (ROIs). Unlike a standard independent components analysis (ICA), which is applied to the entire fMRI time series, the eICA method is applied only to the event-related time courses at each voxel (an estimate of the event-related signal at each voxel is first obtained by deconvolution of the observed fMRI signal with the observed EEG event timing), which means that only a small number of components are generated that are all explicitly related to the event of interest. Event-related ICA can be applied to data from individual subjects and also to group data using a temporal concatenation approach. We previously demonstrated that the eICA method, when applied to EEG-fMRI data acquired from a group of patients with Benign epilepsy with centro-temporal spikes (BECTS), provided better performance than a standard event-related analysis and a linear deconvolution approach, with a better detection rate in single-subject analyses (73 vs. 53%) and only event-related ICA finding significant group-level activation (Masterton et al., 2013b).

A key element of the eICA is the initial deconvolution. However, the stability of the deconvolution can be compromised by various sources of structured noise (Biswal et al., 1996; Friston et al., 1996; Glover et al., 2000) present in the fMRI signal. These include rapid and slow head movements, physiological activity (breathing and heartbeat) and potential acquisition artifacts. Data driven techniques, especially ICA, are increasingly being employed to separate signal and noise in conventional fMRI data (Thomas et al., 2002; Kochiyama et al., 2005; McKeown et al., 2006; Perlbarg et al., 2007; Stevens et al., 2007; Calhoun et al., 2008; Tohka et al., 2008; Sui et al., 2009; Beckmann, 2012; Kundu et al., 2012; Bhaganagarapu et al., 2013; Salimi-Khorshidi et al., 2014). However, in the context of EEG-fMRI studies in epilepsy, the interpretation of the results from an application of ICA can be difficult as it may produce more than a hundred different components per subject with the majority of these likely having no relationship to the EEG event of interest (Rodionov et al., 2007; LeVan et al., 2010).

To address this we developed a strategy for the automated objective identification of artifactual components from an ICA, that we have dubbed a Spatially Organized Component Klassificator (SOCK) (Bhaganagarapu et al., 2013). The primary objective of SOCK is to distinguish noise from true activation without rejecting the latter. SOCK automatically classifies ICs into one of two categories; artifact or unlikely artifact. It does so using spatial measures likely to indicate motion, physiological noise, or machine or undetermined noise. SOCK was shown to successfully remove artifactual components, without rejecting true activation in resting state data (Bhaganagarapu et al., 2013). Unlike existing automatic classifier methods which are primarly dependent on training data to inform classification (De Martino et al., 2007; Tohka et al., 2008; Salimi-Khorshidi et al., 2014) or require querying a public database (Sochat et al., 2014), SOCK is a standalone, automated and objective method that does not require the user to train the algorithm. It is able to identify a high proportion of artifact-related ICs without removing components that are likely to be of neuronal origin (Bhaganagarapu et al., 2013).

In this paper, we extend the eICA procedure by utilizing SOCK to automatically de-noise fMRI data from an EEG-fMRI acquisition prior to running eICA. As we are de-noising the entire fMRI time series prior to the eICA, we hypothesize that this approach has the potential to be more sensitive than eICA alone. The use of an automated de-noising procedure like SOCK in the context of eICA is a novel methodology and to our knowledge has not been investigated previously. We demonstrate the effectiveness of SOCK by comparing the extent of activation obtained in a standard eICA analysis of EEG-fMRI data with and without the use of SOCK for 14 patients with rolandic epilepsy who exhibited stereotypical IEDs arising from a focus in the rolandic fissure.

## 2. Methods

### 2.1. Methods overview

**Figure 1:**
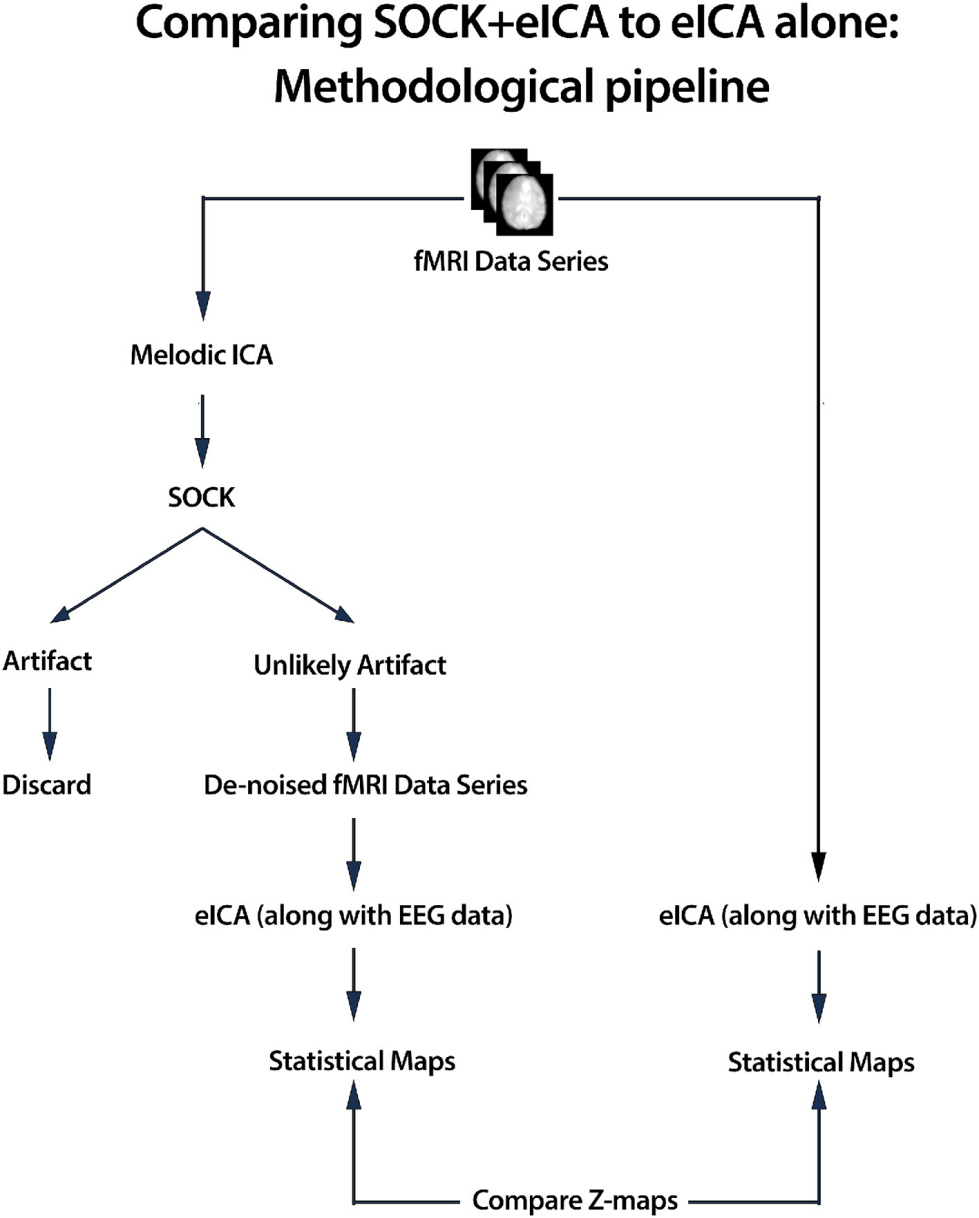
**We assess the performance of SOCK by comparing the activation obtained in an eICA analysis with and without the use of SOCK (for both group and individual studies)**. In the with-SOCK processing stream, ICA was applied to pre-processed fMRI data yielding spatial component maps with associated time courses and power spectra, SOCK automatically classified ICs into one of two categories; artifact or unlikely artifact. Rejecting all ICs classified into the artifact category, a de-noised fMRI data series is formed that is then processed with eICA.

SOCK is applied to de-noise fMRI data prior to event-related ICA. An overview of the de-noising process is given below (see also Figure 1) and more detail is provided in the sections that follow.

1. ICA was applied to the pre-processed fMRI data (see Section 2.6) using MELODIC (Beckmann and Smith, 2004), yielding both thresholded and unthresholded ICs and associated time courses and power spectra^1^.
2. ICs were classified into one of two categories using SOCK: artifact or unlikely artifact.
3. All ICs classified into the artifact category were discarded and a de-noised fMRI data series was constructed with only the unlikely artifact ICs.
4. An event-related ICA (eICA) was preformed using the de-noised fMRI data (along with EEG).
  The above process was performed for both group and individual studies.

### 2.2. ICA decomposition

In the with-SOCK processing stream, ICA is employed to decompose the 4D fMRI time series into a linear combination of spatially independent component maps with an associated time-course (McKeown et al., 1998; Hyvärinen, 1999). In practice this decomposition is usually too computationally expensive to perform on raw fMRI data, so a preliminary data reduction step using principal components analysis is applied prior to ICA. Several freely available software packages are available to perform this preprocessing and decomposition; we used MELODIC which is part of the FSL package (Beckmann and Smith, 2004). The output is a set of spatial maps with associated time courses and power spectra. These then form the input for the automatic classifier, SOCK.

### 2.3. Classification of ICs using SOCK

SOCK classifies ICs using features likely to indicate motion, physiological noise, or machine or undetermined noise. The algorithm is described in detail elsewhere (Bhaganagarapu et al., 2013) and our implementation is freely available at www.brain.org.au/software. Briefly, individual slices in each IC are assessed for:

1. Smoothness: contributions of low and high spatial frequency content, to detect components with a large number of isolated very small clusters or isolated voxels (i.e., a spotty appearance).
2. Edge activity: extent of activity in an edge mask.
3. Ventricular activity: extent of activity in a Cerebrospinal fluid (CSF) mask.
4. Temporal Frequency Noise (TFN): the power in temporal frequency beyond 0.08 Hz.

Based on these measures and with the assistance of k-means clustering, ICs dominated by artifact are classified into an Artifact category and all other ICs (i.e., those containing possible neuronal signal) into an Unlikely Artifact category.

The SOCK procedure was implemented in MATLAB (R2010b, The MathWorks, Natick, MA, USA). Source code of our implementation of the method is available at http://www.brain.org.au/software.

### 2.4. Constructing de-noised data

After SOCK classification, all ICs classified in the artifact category are discarded from the fMRI data set and a de-noised fMRI data set is assembled from the remaining components. This is done via the FSL function, fsl_regfilt (with the ‘-a’ aggressive filtering option). The de-noised fMRI data along with the original EEG timings are then input to an eICA analysis.

### 2.5. Event-related independent components analysis (eICA)

The eICA method, described in detail elsewhere (Masterton et al., 2013b), can be applied at either an individual or group level. In brief, eICA uses two separate steps to identify events observed in the EEG: firstly, a linear deconvolution (via GLM) provides an estimate of the event-related BOLD response at each voxel in the brain in a time-window spanning from 30 s before to 30 s after the event onset. The deconvolution does not assume any particular response shape and allows for changes occurring before the event onset. ICA is then used to separate the estimated event-related fMRI signal changes into a small number of spatial maps and associated time-courses that summarize the timing of activity within different spatial sources.

To estimate the event-related response across the group, the ICA decomposition was performed upon temporally concatenated data (Calhoun et al., 2009); note that in this context the event related responses (rather then the original fMRI time series) were concatenated. This provided a common set of spatial maps for each group with subject specific time courses. Components of interest were identified as those exhibiting activity in the vicinity of the ipsilateral rolandic region (Masterton et al., 2013b).

The eICA procedure was implemented in MATLAB (R2010b, The MathWorks, Natick, MA, USA) using the SPM8 software (Wellcome Department of Cognitive Neurology, http://www.fil.ion.ucl.ac.uk/spm) to perform the GLM parameter estimation, and the FastICA and ICASSO (Hyvärinen, 1999; Himberg et al., 2004) toolboxes to perform the ICA decomposition. The resulting spatial maps were transformed into z-statistics maps by fitting a mixture model to the data (see Masterton et al., 2013b for more details). This eICA procedure was applied to EEG-fMRI data in separate analyses with and without de-noising the fMRI data via SOCK.

### 2.6. fMRI data

#### 2.6.1. Subjects

The fMRI data used for this study was the same as that previously studied with eICA (without SOCK) and described in detail by Masterton et al. (2013b). We summarize key subject details below and in Table 1. Data from fourteen patients with typical BECTS, recruited for EEG-fMRI from the Royal Childrens Hospital, Monash Medical Centre and Austin Hospital in Melbourne, Australia, are included in the analysis. One patient had independent left and right-sided CTS; the remainder had unilateral discharges—this provided a total of fifteen different events for study. More detail on the patient cohort is provided in Lillywhite et al. (2009). A representative EEG recording of CTS discharges in the MRI scanner is also provided in Masterton et al. (2010). This cohort was chosen because the previously published eICA could be used as a gold standard when assessing the performance of SOCK to de-noise fMRI data. This study had approval from the Human Research Ethics Committee at each recruiting hospital and all subjects (or their parents) provided written informed consent.

**Table 1:** **Patient details**.

**Subject ID**	**Gender**	**Age at study**	**CTS laterality**	**Number of events**
1	M	6	Right	509
2	M	7	Left	527
3	M	7	Left	38
4	F	9	Left	622
5	M	9	Left	428
			Right	434
6	M	9	Right	67
7	F	9	Left	106
8	M	9	Left	348
9	M	10	Right	670
10	M	10	Right	285
11	F	10	Right	257
12	M	10	Left	158
13	M	11	Right	134
14	M	13	Right	15

#### 2.6.2. Data acquisition

The patients underwent 30 min of simultaneous EEG and fMRI scanning. fMRI images were acquired in a 3T GE Signa LX scanner (General Electric, Milwaukee, WI, USA) using a BOLD-weighted gradient-recalled echo-planar imaging sequence (*TR* = 3 s; *TE* = 40 ms; FOV = 24 × 24 cm; 128 × 128 matrix; 25 interleaved 4 mm slices with 1 mm gap). In three studies (Subjects 3, 7, and 9 in Table 1) a slightly different fMRI acquisition was used (*TR* = 3 s; *TE* = 40 ms; FOV = 22 × 22 cm; 64 × 64 matrix; 35 interleaved 3.2 mm slices with 0.2 mm gap).

Simultaneous EEG was acquired during fMRI scanning using an MR-compatible EEG system (developed in-house) with scalp electrodes positioned in the standard 10–20 locations and filtering to remove the effect of cardioballistic and motion artifacts (Masterton et al., 2007). The patients' EEG was reviewed by experienced electroencephalographers according to the guidelines developed in our group (Flanagan et al., 2009) and the timing of all identified CTS was recorded.

#### 2.6.3. Data analysis

Image conversion was performed using iBrain (Abbott and Jackson, 2001), preprocessing and statistical analysis utilized SPM8 with the aid of the iBrain Analysis Toolbox for SPM (Abbott et al., 2011; www.brain.org.au/software). Preprocessing included temporal alignment of slices within each volume to the first slice, rigid-body spatial realignment to correct for subject motion, spatial normalization to a symmetric template and spatial smoothing with a Gaussian kernel (FWHM = 8 mm). The symmetric template was created specifically for this patient group using SPM8 software by normalizing each subject's brain to MNI space, averaging these images together (along with a left-right flipped version of each image), and then smoothing with an 8 mm Gaussian filter (Wilke et al., 2002). To enable grouping of data between subjects with left and right-sided CTS, the data from subjects with right-sided CTS were flipped in the left-right direction prior to group analysis.

## 3. Results

### 3.1. ICA analysis and SOCK classification

MELODIC ICA yielded an average of 114 components per subject (range: 57–285). SOCK classified between 27 and 53% of each subject's components as artifact (mean 41%). These ICs were discarded to construct a de-noised fMRI data set for each subject. See Table 2 for summary details of the ICA decomposition and the SOCK classification for all 14 subjects.

**Table 2:** **ICA decomposition and the SOCK classification for 14 patients who underwent an EEG-fMRI study as described in Section 2.6.2**.

**Subject ID**	**No. of ICA components**	**SOCK classification Artifact**	**artifact**	**% of rejected ICs**
1	97	43	54	44
2	75	29	46	39
3	81	31	50	38
4	105	45	60	43
5	57	30	27	53
6	122	50	72	41
7	80	29	51	36
8	161	56	105	35
9	154	51	103	33
10	108	49	59	45
11	285	78	207	27
12	99	44	55	44
13	120	50	70	42
14	106	44	62	42

*SOCK classified between 27 and 53% of each subject's components as artifact (mean 41%)*.

### 3.2. Individual subject event-related analysis

Fifteen individual analyses were performed for eICA, each with and without the use of SOCK to de-noise fMRI data. This included two analyses for the patient (Subject 5) that had independent left and right-sided CTS, which were analyzed as separate events. The results are summarized in Table 3.

**Table 3:** **A summary of the results of the individual analyses comparing the number of ICs yielded from an eICA with and without the use of SOCK prior to eICA**.

**Subject ID**	**eICA**		**SOCK+eICA**	
	**No. of components**	**Rolandic component?**	**No. of components**	**Rolandic component?**
1	8	✓	1	✓
2	2	✓	2	✓
3	6	-	6	-
4	7	✓	10	✓
5 (left CTS)	7	✓	6	✓
5 (right CTS)	6	✓	9	✓
6	7	-	5	-
7	10	✓	10	✓
**8**	**2**	**-**	**5**	**✓**
9	11	✓	4	✓
**10**	**6**	**-**	**6**	**✓**
11	5	✓	5	✓
12	9	✓	8	✓
13	10	✓	9	✓
14	9	✓	9	✓

*The rolandic component column indicates whether an eICA component was visually identified with a BOLD signal change in the peri-rolandic region. Rows in bold indicate subjects where activation in the vicinity of the ipsilateral rolandic region was identified when using eICA+SOCK but not when using eICA alone*.

### 3.3. eICA (without SOCK)

eICA estimated an average of 7 (range: 2–11) different components for each analysis. In 11 out of 15 analyses (73%) at least one component was identified showing activity in the vicinity of the ipsilateral rolandic region.

### 3.4. eICA (with SOCK)

After de-noising the fMRI data with SOCK, eICA estimated an average of 6 (range: 1–10) different components for each analysis. In 13 out of 15 analyses (87%) at least one component was identified showing activity in the vicinity of the ipsilateral rolandic region.

**Figure 2:**
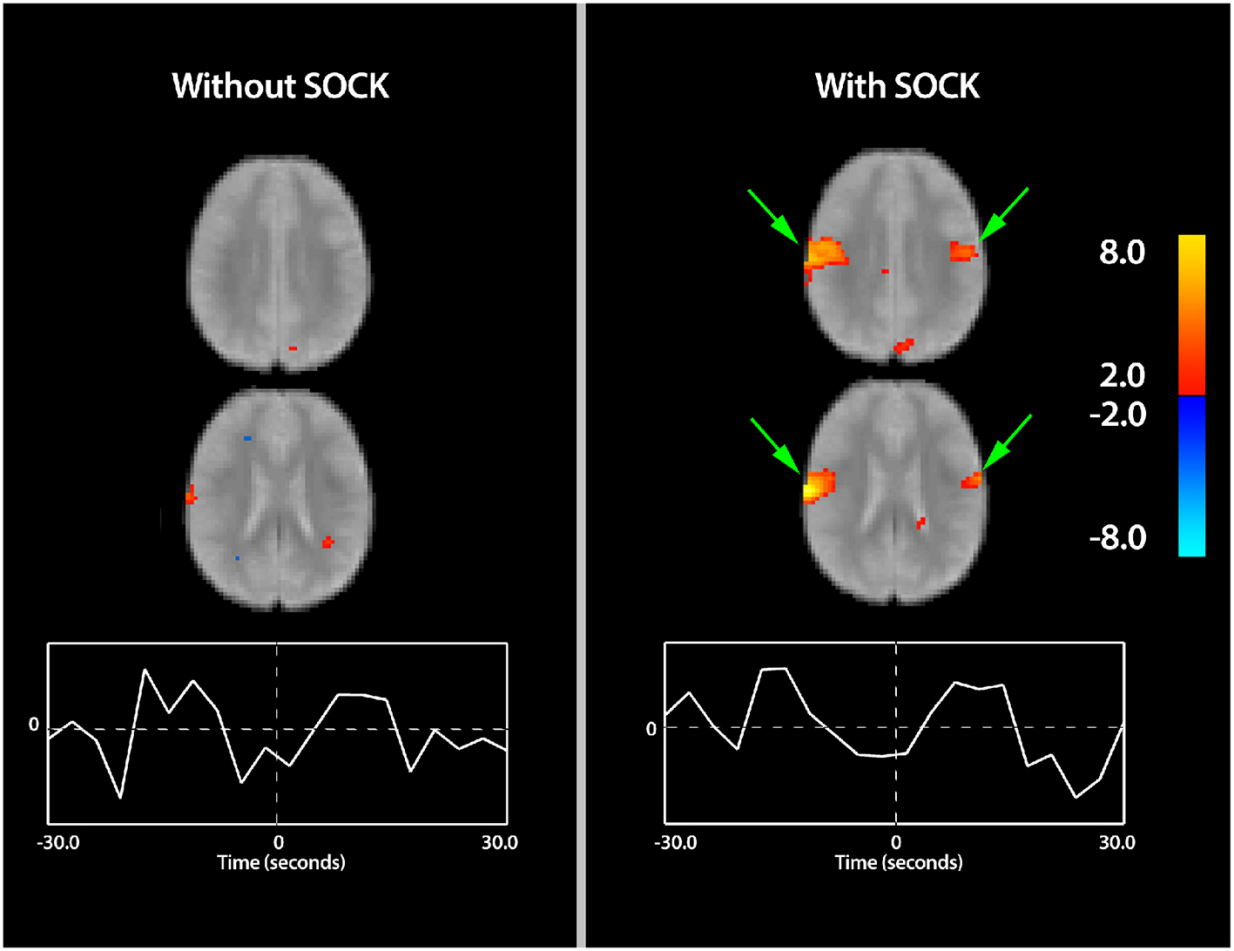
**Spatial maps (thresholded at *p* < 0.05) with time courses for subject 11 indicating the differences in activation with and without the use of SOCK (left and right columns respectively)**. Activation is overlaid onto a mean functional image for this subject. Warm and cool colors indicate respectively a positive or negative correlation with the component time course. Arrows in green show areas of increased activation within the region of interest when SOCK was used. Furthermore, the shape of the time-course after applying SOCK is also qualitatively smoother then prior to using SOCK. The zero time-point, indicated by the vertical dotted line in the center of the time-course plot, represents the onset time of the EEG discharge.

Figure 2 displays sample slices and time-courses of the rolandic component derived from an eICA of subject 11, with and without SOCK. Activation is overlaid onto a mean functional image for that subject. Comparison of the left (without SOCK) and right (with SOCK) columns shows more robust activation in the area of interest after de-noising the data with SOCK and additional activation on the contralateral side (see green arrows). Furthermore, the shape of the time-course after applying SOCK is also qualitatively smoother.

**Figure 3:**
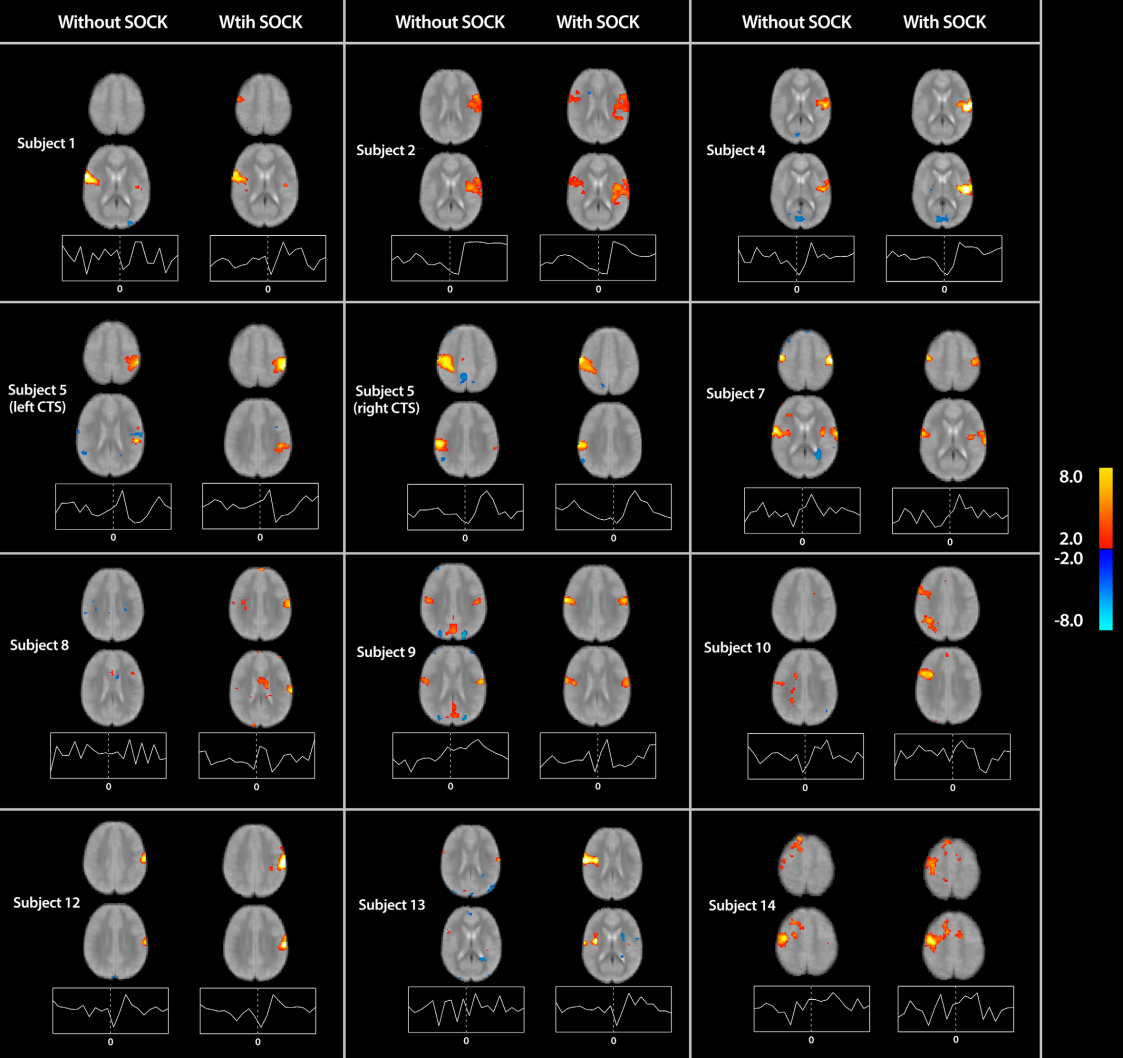
**Spatial maps (thresholded at *p* < 0.05) with time courses for all subjects (except subject 11 already shown in Figure 2) indicating the differences in activation with and without the use of SOCK (left and right columns respectively)**. Activation is overlaid onto a mean functional image for each subject. Warm and cool colors indicate respectively a positive or negative correlation with the component time course. The time course axes are similar to Figure 2. The zero time-point, indicated by the vertical dotted line in the center of each time-course plot, represents the onset time of the EEG discharge. Two subjects (8 and 10) yielded activation in the vicinity of the ipsilateral rolandic region when analyzed with eICA after de-noising with SOCK but not when analyzed by eICA alone. Furthermore, the shape of the time-course after applying SOCK is also qualitatively smoother then prior to using SOCK.

The associated spatial maps (with and without SOCK) and time courses for all remaining subjects are provided in Figure 3.

In two of these analyses (Subjects 8 and 10) activation in the vicinity of the ipsilateral rolandic region was identified when analyzed with eICA after de-noising with SOCK but not when analyzed by eICA alone (see Figure 3). The shape of the time-courses after applying SOCK for these subjects was also qualitatively consistent with the other subjects' peri-rolandic component time courses.

Furthermore, using SOCK prior to running an eICA has qualitatively decreased the noise in both the spatial maps and time courses. For example, the spatial maps for Subjects 9 and 13 (Figure 3) are observed to have little or no activation on the edge of the brain and in the CSF after applying SOCK. In addition, the time courses are observed to follow a BOLD response more consistent with the other subjects.

### 3.5. Group event-related analysis

### 3.6. eICA (without SOCK)

**Figure 4:**
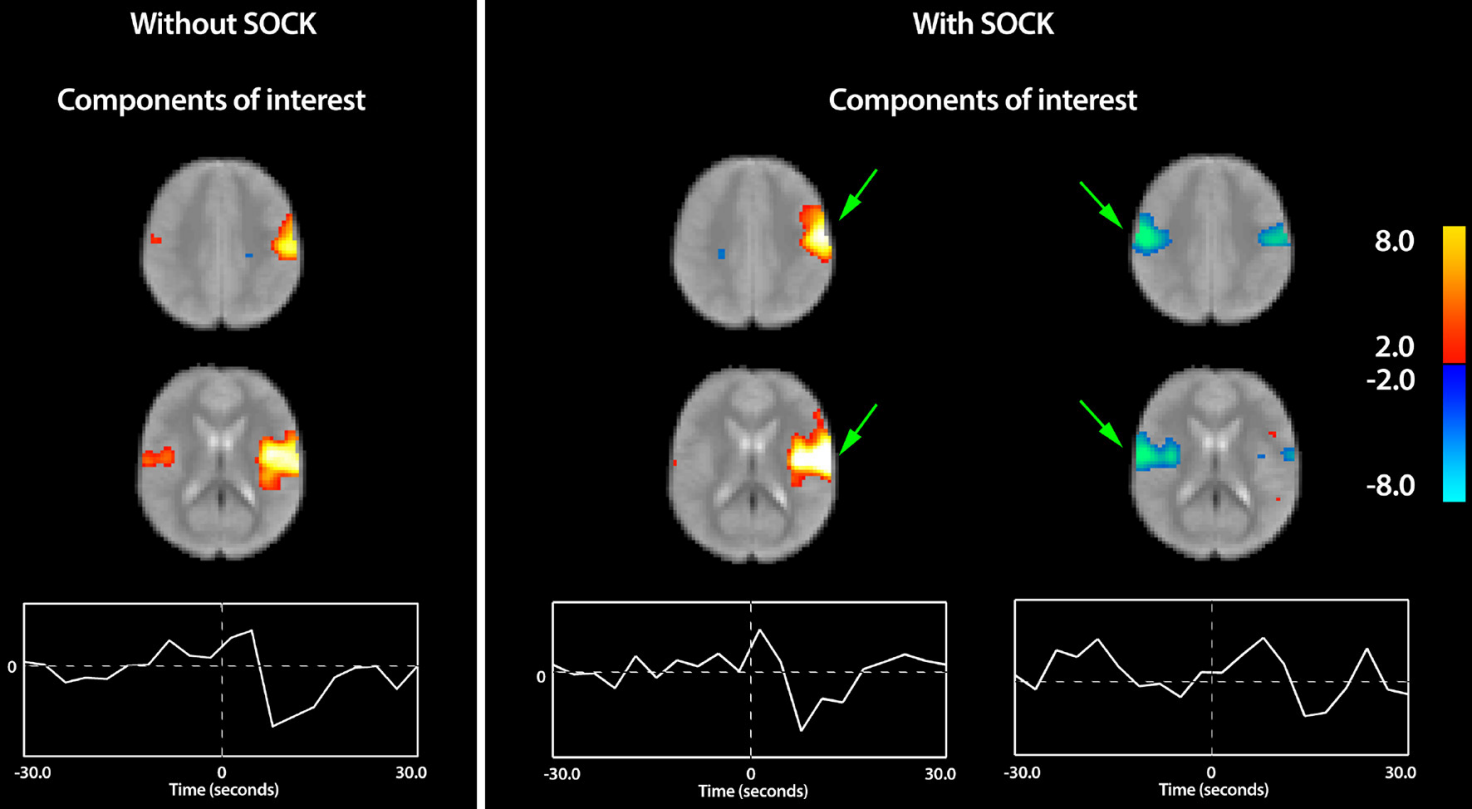
**The group eICA result**. **Left panel**: Only a single group component of interest was found for the eICA only analysis (Without SOCK), which is dominated by activity low in the ipsilateral post-central gyrus, extending along its opercular surface into the lateral fissure. A much smaller region was also seen on the contralateral side. **Right panel**: The SOCK+eICA analysis separated these regions into distinct components with different time-courses and revealed a substantially larger extent of de-activation on the contralateral side (see far right component) that appears to have a time course somewhat delayed from the ipsilateral-only component. Arrows in green highlight areas of substantial improvement when SOCK was used. The components are displayed as z-statistic maps thresholded at *p* < 0.05 corrected for multiple comparisons and overlaid upon the group-mean fMRI image. The time-course at the bottom represents the average modulation of this network across all the subjects i.e., the event-related impulse response function. The zero time-point, indicated by the vertical dotted line in the center of the plot, represents the onset time of the EEG discharge. Warm and cool colors indicate respectively a positive or negative correlation with the component time course.

eICA group analysis (without SOCK) estimated 14 different components out of which a single CTS-related component of interest was identified low in the ipsilateral post-central gyrus, extending along its opercular surface into the lateral fissure. A much smaller activation was also seen on the contralateral side (see “Without SOCK” panel in Figure 4). It is important to note that the term “activation” used here is defined based upon the direction of signal change near the EEG event onset at time 0; however if the haemodynamic contribution to the response is assumed canonical then the larger than canonical post event dip suggests there is substantial neuronal deactivation following an initial smaller positive neuronal activation event in this component.

### 3.7. eICA (with SOCK)

The SOCK+eICA analysis yielded 15 different components out of which two CTS-related components of interest were identified; (1) low in the ipsilateral post-central gyrus, extending along its opercular surface into the lateral fissure (also observed in eICA without SOCK analysis) and (2) a bilateral component containing de-activation in both the ipsilateral region above and contralaterally (see Figure 4).

## 4. Discussion

In this paper we have demonstrated the superiority of SOCK+eICA compared with eICA alone for mapping functional brain activity associated with epileptic spikes. It has previously been demonstrated that that eICA is superior to conventional event-related analyses when the BOLD response does not closely match the canonical haemodynamic response function (HRF) (Masterton et al., 2013b). Taken together, our results suggest that SOCK+eICA should replace eICA alone as the preferred method for such analyses.

The centro-temporal spikes of Rolandic epilepsy served as a good test case for our analysis methodology, as it is known that the BOLD response does not well match the standard HRF. This is in part due to neuronal activity associated with the spikes being detectable with fMRI before activity becomes sufficiently widespread and synchronized to manifest as a spike visible on the EEG, and in part due to a larger post-spike undershoot (Masterton et al., 2010, 2013b). The eICA procedure provides a less constrained approach than a GLM incorporating a conventional HRF model, however this flexibility comes at the cost of lower power (increased susceptibility to noise). Whilst the event-related nature of the eICA approach provides a much stronger constraint than conventional ICA on the full fMRI time series, the eICA method is still susceptible to noise, particularly in the initial deconvolution step. Temporally non-stationary noise would be expected to increase the heterogeneity of the raw signal response associated with events, making deconvolution more challenging, and spatial non-stationary of the noise would be expected to increase heterogeneity of the derived event-related responses across voxels. This would then deleteriously affect the performance of the subsequent ICA of the event-related responses. Thus using a procedure which removes a substantial quantity of noise from the input data may improve the end result. The SOCK procedure is a standalone, automated and objective method which is able to remove a substantial fraction of noise without removing biologically interesting signal (Bhaganagarapu et al., 2013). The results of the present study indicate in practice the improvement can be substantial when SOCK is used to de-noise fMRI data prior to eICA.

Applying SOCK+eICA to the existing EEG-fMRI of our BECTS cohort has improved the confidence in the initial results of Masterton et al. (2010) and Masterton et al. (2013b) (i.e., that the centro-temporal spikes arise from low in the ipsilateral post-central gyrus, extending along its opercular surface into the lateral fissure), with two of the previously negative-result individuals now showing activity in this region. There now remain just two individuals with negative results: These two subjects (3 and 6 in Table 1) along with subject 14 registered the smallest number of events compared to all other subjects. The lower the event count, the less power one has to detect an effect (Flanagan et al., 2009).

In these BECTS subjects the epileptic spikes were unilateral during the imaging session. Homologous regions of cortex are connected via fibers projecting through the corpus callosum and typically inhibit neural activity in the contralateral hemisphere. The new SOCK+eICA group analysis reveals a more complex response than previously evident, with initial ipsilateral activity, followed by a more extensive bilateral pattern of deactivation (i.e., the time-course of the deactivation component displays a later rise and peak compared to the ipsilateral-only component). We would interpret the new finding as distinguishing the activation of the ipsilateral cortex during epileptiform events, and a later bilateral decrease in activity in response to this activation.

## 5. Conclusion

We have demonstrated a novel application of our ICA classifier, SOCK, to de-noise fMRI prior to an event-related ICA in patients with rolandic epilepsy. The procedure outlined in this paper harnesses the advantage of both techniques: (1) SOCK de-noises fMRI in an objective and automated manner utilizing the entire fMRI time-series. (2) eICA utilizes the EEG information to derive event-related responses which are input into an ICA, thus constraining the final eICA decomposition to a small number of components time-locked to the events of interest. The use of SOCK increased power to detect activity of interest in both individual and group analyses.

### Conflict of interest statement

The authors declare that the research was conducted in the absence of any commercial or financial relationships that could be construed as a potential conflict of interest.

## Footnotes

^1^Temporal information expressed in the frequency domain. This is done mathematically by taking the discrete Fourier Transform of the time course.
